# Anterior Optic Nerve Head Perfusion is Dependent on Adjacent Parapapillary Choroidal perfusion

**DOI:** 10.1038/s41598-019-47534-5

**Published:** 2019-07-29

**Authors:** Kyoung Min Lee, Joon Mo Kim, Eun Ji Lee, Tae-Woo Kim

**Affiliations:** 1 Department of Ophthalmology, Seoul National University College of Medicine, Seoul, Korea; 2 Department of Ophthalmology, Seoul National University Boramae Medical Center, Seoul, Korea; 3 Department of Ophthalmology, Kangbuk Samsung Hospital, Sungkyunkwan University School of Medicine, Seoul, Korea; 4 Department of Ophthalmology, Seoul National University Bundang Hospital, Seongnam, Korea

**Keywords:** Optic nerve diseases, Retina

## Abstract

Recent studies reported that parapapillary microvascular dropout (MvD) was significantly associated with glaucoma and glaucoma progression. To understand the clinical relevance/importance of MvD, it is essential to know the exact vascular anatomy of optic nerve head (ONH). Although it is known that parapapillary choroid and the deep ONH structure including prelaminar tissue are both supplied by branches of short posterior ciliary artery, it remains controversial whether parapapillary choroid provides a major contribution to the prelaminar tissue perfusion. This study investigated perfusion within and around the ONH using indocyanine green angiography. Thirty-three eyes from 33 patients with primary open-angle glaucoma and 10 eyes from 10 normal subjects were included. The temporal sequence of dye appearance in various tissues was analyzed. We also sought the microvessels directly responsible for blood supply to the prelaminar tissue. The perfusion of the prelaminar tissue, which occurred in a sectoral fashion, was dependent on the dye appearance in the adjacent parapapillary choroid. In addition, microvessels crossing over the optic disc margin from the parapapillary choroid to the ONH were found. The findings suggest that the centripetal flow from the parapapillary choroid is an important source of prelaminar tissue perfusion.

## Introduction

Glaucoma is a multifactorial disease^1^. Although intraocular pressure (IOP)-induced stress is considered to be the most important risk factor^2–4^, other factors including microvascular compromise within the optic nerve head (ONH) are also thought to be involved in the pathogenesis of glaucomatous optic neuropathy^5–8^. Recent studies demonstrated microvascular dropout (MvD) in the parapapillary deep layer of primary open angle glaucoma (POAG) eyes using optical coherence tomography angiography (OCTA)^9,10^. The MvD was shown to correspond to a perfusion defect as assessed in indocyanine green (ICG) angiography (ICGA) suggesting that the MvD reflects true perfusion impairment in the choroidal layer^11^. Since the parapapillary choroid and the deep ONH are both supplied from branches of short posterior ciliary artery (SPCA), the MvD may indicate an important vascular event that may be relevant with glaucoma development and progression. Indeed, faster progression of retinal nerve fiber layer thinning^12^, association with early parafoveal visual field defects^13^ and worse visual field at the same level of RNFL thickness^14^ were demonstrated. However, little is known yet about the pathogenic mechanism how the MvD develops and in what way it is related with glaucoma development and progression.

Short posterior ciliary artery (SPCA) supplies the prelaminar tissue and the lamina cribrosa of the ONH^15^. However, controversy remains regarding the blood supply to the prelaminar tissue at the microvascular level. Some reports contended that the peripapillary choroid is the primary supply to the prelaminar region of the ONH^15,16^. In contrast, others have asserted that parapapillary choroid had no or minimally significant role in the blood supply of the ONH^17–19^.

Given the association of MvD with glaucoma, it is important to establish the microvascular anatomy of the prelaminar tissue supply. Such knowledge may provide a platform to enhance the insight on the relevance of MvD with glaucoma. The purpose of this study was to evaluate the anterior ONH blood supply using ICGA which enables high resolution imaging of the microvasculature.

## Results

Initially, 46 eyes were included in the study, of which 3 were excluded due to poor ICGA image quality, leaving a final sample of 33 POAG eyes and 10 healthy eyes. The demographics of the included subjects are given in Table 1. The measurement of filling time in each vascular structure showed excellent interobserver agreement. The intracorrelation coefficients (ICC) were 0.999 (95% CI, 0.999–1.000) for parapapillary choroidal arteriole, 0.992 (95% CI, 0.963–0.998) for cilioretinal artery, 0.999 (95% CI, 0.999–1.000) for microvessels crossing from the choroid to the ONH, 0.998 (0.994–0.999) for central retinal artery (CRA), and 0.997 (95% CI, 0.995–0.999) for peak intrapapillary filling.

**Table 1:** Demographic profiles of the study subjects.

Characteristic	POAG (n = 33)	Normal subjects (n = 10)	*P*-value*
Age (years)	52.8 ± 11.5	50.8 ± 12.7	0.635
Males:females (*n*:*n*)	10:23	4:6	0.566
Refraction (diopters)	−2.22 ± 2.81	−1.41 ± 2.82	0.437
Untreated IOP (mmHg)	16.8 ± 4.3	13.3 ± 3.3	0.024
IOP_ICGA_ (mmHg)	12.2 ± 1.9	13.3 ± 3.3	0.392
CCT (µm)	544 ± 46	555 ± 18	0.216
Axial length (mm)	24.7 ± 1.6	24.3 ± 1.2	0.556
Mean deviation (dB)	−6.42 ± 5.93	−0.32 ± 0.47	<0.001
Average RNFL thickness (µm)	75.8 ± 14.1	104.3 ± 10.2	<0.001
Systolic BP (mmHg)	123 ± 10	122 ± 3	0.685
Diastolic BP (mmHg)	74 ± 9	74 ± 7	0.697

Data are mean ± SD values.

^*^Calculated using the Mann-Whitney U test for continuous variables, and chi-square test for categorical variables.

POAG = primary open angle glaucoma, IOP = intraocular pressure, IOP_ICGA_ = IOP at the time of indocyanine green angiography, CCT = central corneal thickness, RNFL = retinal nerve fiber layer, BP = blood pressure.

### ONH filling

**Figure 1:**
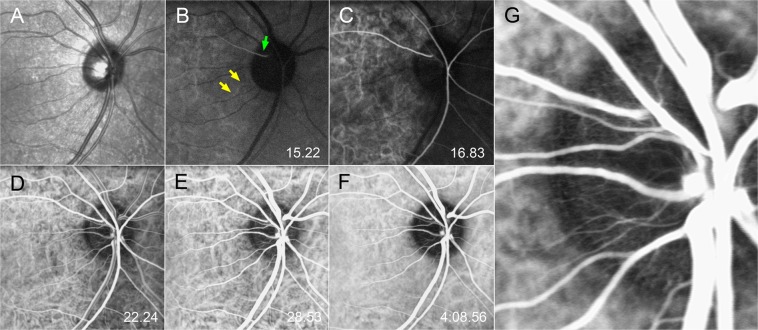
Serial indocyanine green angiography (ICGA) images on the right eye of a normal subject. (**A**) Infrared fundus image acquired before the ICGA. (**B–F**) Serial ICGA images. The elapsed time since dye injection is indicated in the lower right corner. (**B**) Dye appears in the parapapillary choroidal arteriole (yellow arrows) and ciliary artery (green arrow). (**C**) Dye filling is seen in the central retinal artery and in the temporal optic nerve head (ONH). (**D**) ONH filling is nearly completed. (**E**) Image obtained at the time of peak ONH filling. (**F**) In the late phase, the ONH area turns into hypolucent. (**G**) Magnified view of the temporal ONH at the time of peak ONH filling shown in. (**E**) Centripetal microvessels are observed at the disc margin.

**Figure 2:**
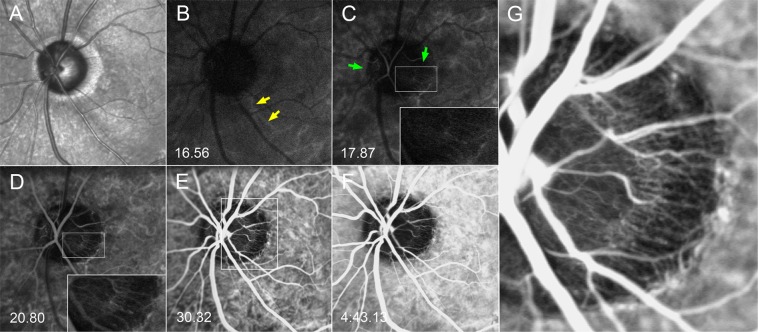
Serial ICGA images on the left eye of a glaucoma patient. (**A**) Infrared fundus image acquired before the ICGA. (**B**) Dye appearance in the parapapillary choroidal arterioles is just visible (yellow arrows). (**C**) Dye filling is seen in the nasal and temporal sectors (green arrows) of the optic nerve head (ONH). Centripetal microvessels supplying the ONH are discernible (inset). (**D**) The dye filling is more clearly seen in the nasal and temporal sectors of the ONH together with the supplying microvessels (inset). However, it is not yet seen in the superior and inferior sectors. Centripetal microvessels are not visible in those regions. (**E**) Image obtained at the time of peak ONH filling. The dye is now seen throughout the ONH, and centripetal microvessels are now visible in all sectors (see the magnified image shown in **G**). (**F**) Late-phase image. The ONH and parapapillary area are now hypofluorescent. (**G**) Magnified view of the temporal ONH at the time of peak ONH filling. Abundant centripetal microvessels are evident between the choroid and the ONH.

**Figure 3:**
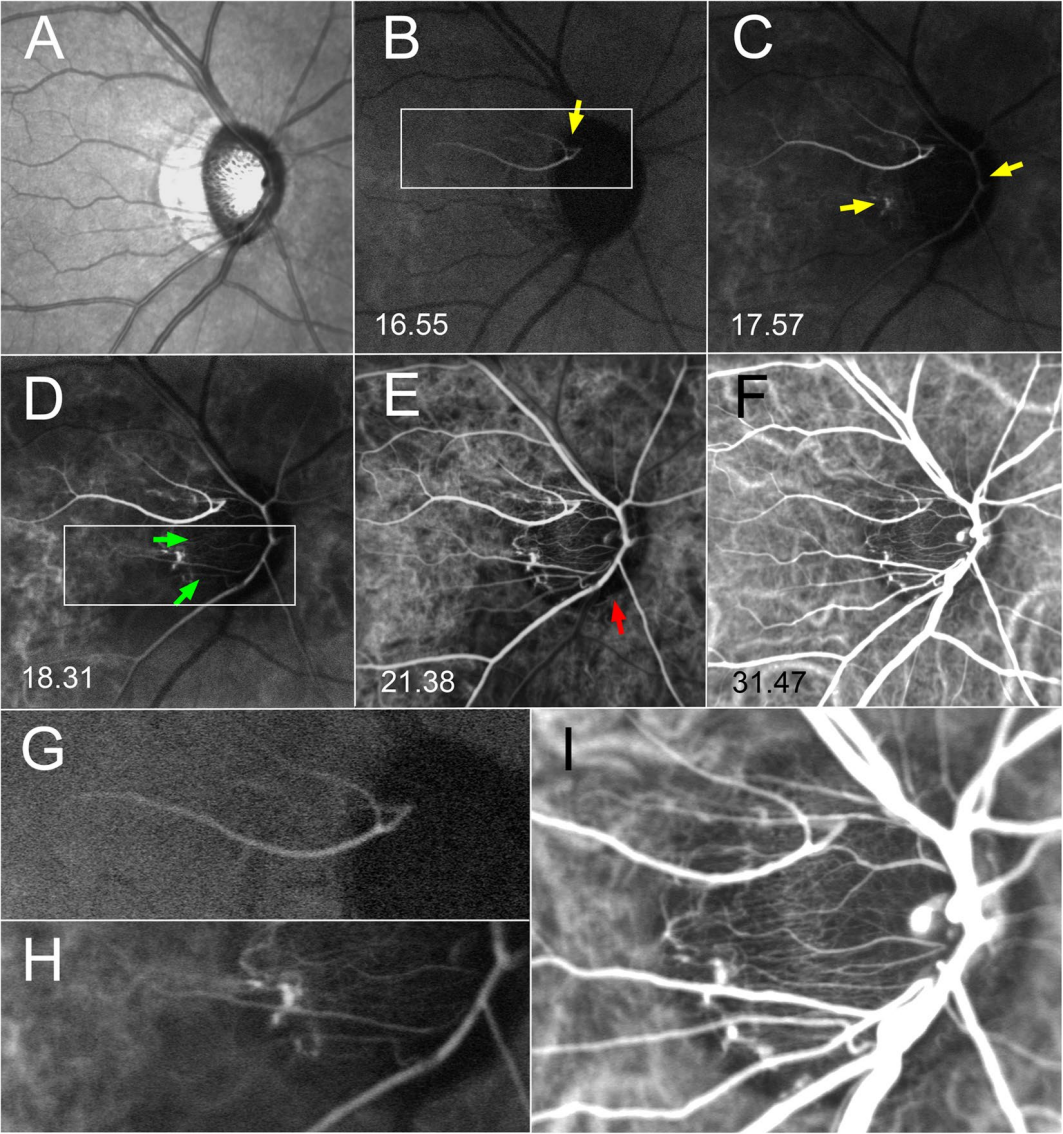
Serial ICGA images on a glaucomatous eye with a cilioretinal artery. (**A**) Infrared fundus image. (**B**) Early-phase image. Dye filling is seen in a cilioretinal artery (yellow arrow), but not within the ONH (see the magnified image shown in **G**). (**C**). Dye filling is also seen in the central retinal artery and parapapillary choroidal arteriole (yellow arrows). Minimal filling is evident in the temporal ONH. (**D**) Dye filling is now noticeable in the temporal ONH (green arrows). In a magnified view of the area marked with box (presented in **H)**, microvessels supplying the ONH are discernible. (**E**) Dye filling is observed in the almost throughout the temporal ONH. However, the inferior neuroretinal rim is not filled (red arrow). (**F**) Image obtained at the time of peak ONH filling. The inferior neuroretinal rim is now also filled. Microvessels from the choroid to the ONH are clearly evident in a magnified view (**I**).

The ONH filling occurred in a sectoral fashion and propagated uniformly from the disc margin to the central retinal vein (i.e., in a centripetal direction) both in normal subjects (Fig. 1, Supplemental video 1) and glaucoma patients (Figs 2 and 3). The time interval between when the dye first appeared within the ONH and peak intrapapillary dye filling was 10.46 ± 2.85 seconds in POAG patients and 9.83 ± 3.43 seconds in normal subjects.

### Temporal relationship between the filling of the optic nerve head and other vascular structures

**Figure 4:**
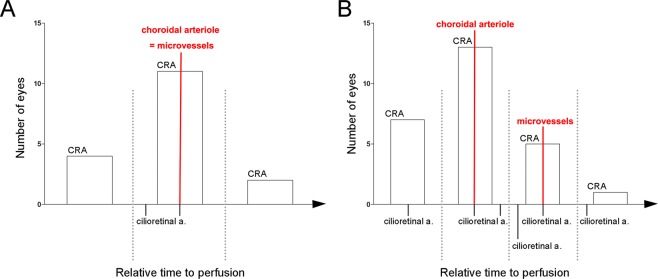
Relative time to perfusion in the branches of ophthalmic artery. Although the order in which dye appeared in various vessels varied markedly among subjects, choroidal arteriole filling was seen at the same angiographic frame (**A**) or earlier (**B)** than the appearance of microvessels crossing over the optic disc margin in all cases. (**A**) Cases where first dye appearances in the parapapillary choroidal arteriole and the microvessels crossing over the optic disc were simultaneous (17 cases in total). In 11 eyes, the central retinal artery (CRA) filling appeared at the same frame with the filling in the parapapillary choroidal arteriole and the microvessels. In 4 eyes, the CRA filled earlier than the latter and vice versa in 2 eyes. (**B**) Cases where first dye appearance in the parapapillary choroidal arteriole was earlier than that in the microvessels (26 cases in total). The first dye appearances in the CRA and cilioretinal artery (cilioretinal a.), in relation with those in the parapapillary choroidal arteriole and the microvessels were highly variable.

**Figure 5:**
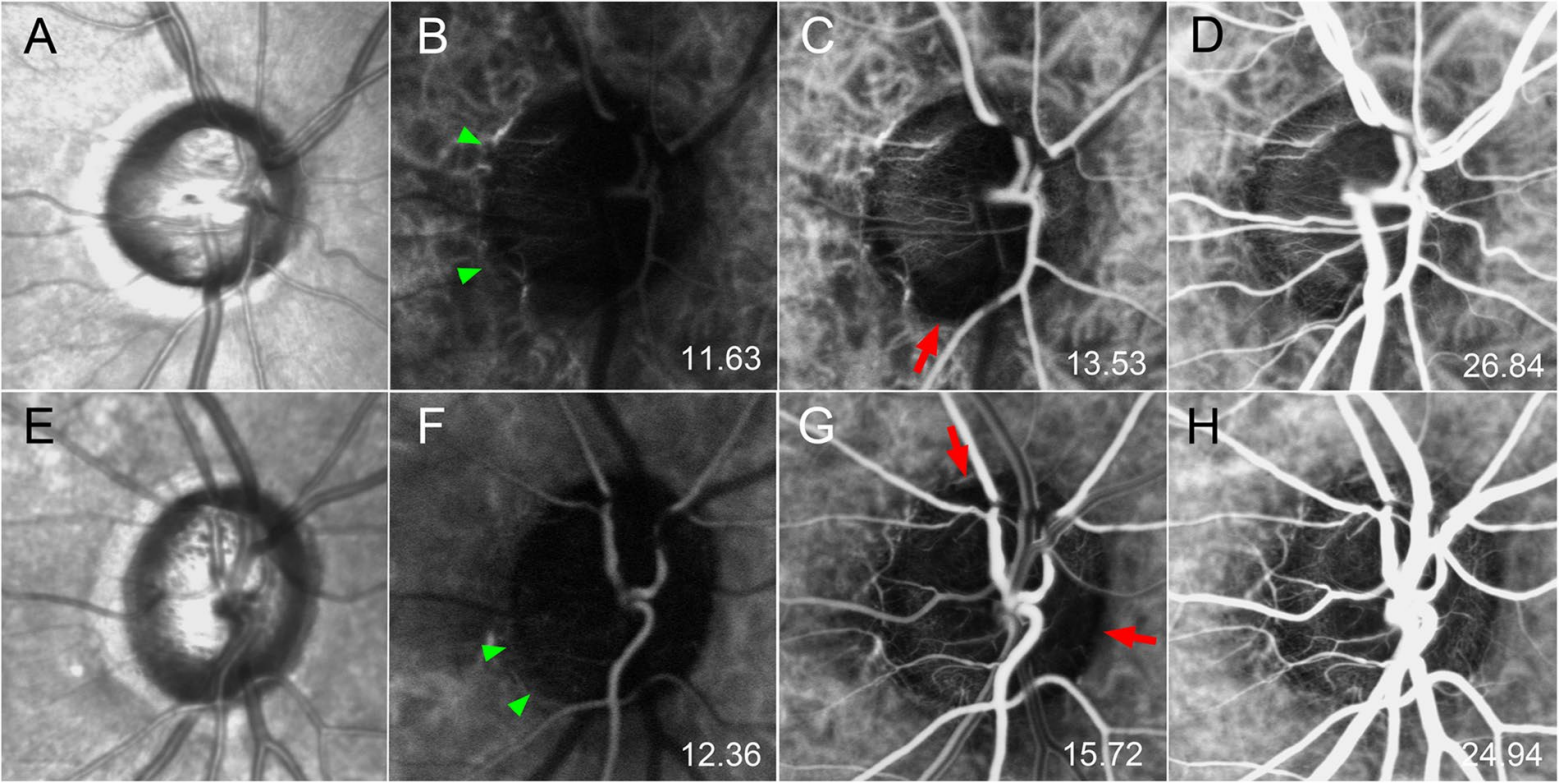
Serial ICGA images of two eyes demonstrating sectoral delayed filling within the ONH. (**A**,**E**) Infrared fundus images. (**B**,**F**) Early-phase image. Microvessels are seen in the temporal ONH (arrowheads); the adjacent choroid is not yet fully filled. (**C**,**G**) Despite dense filling in the adjacent choroid, sectoral nonperfusion is observed within the ONH together with the absence of supplying microvessels (arrows). (**D**,**H**) The ONH is now fully filled.

The order in which dye appeared in various vessels varied markedly among subjects (Fig. 4). CRA, cilioretinal artery, and parapapillary choroidal arteriole were filled without a regular order. It contrasted to parapapillary choroidal arteriole filling being universally seen at the same angiographic frame (Fig. 4A) or earlier (Fig. 4B) than the appearance of microvessels crossing over the optic disc margin. Regions of the ONH were not perfused until the visualization of those microvessels (Figs 1–3, Table 2). The microvessels appeared at the early stage of ONH filling, followed by diffuse filling of the adjacent parapapillary choroid, which probably represented the filling of the choriocapillaris. Occasionally, sectoral delayed filling was found (i.e., the microvessels were not evident even after the diffuse filling of the adjacent parapapillary choroid; Fig. 5). In those cases, the adjacent ONH filling was also delayed until the visualization of microvessels crossing over the disc margin (Fig. 5).

**Table 2:** Times between when dye first appeared in various vascular tissues and in the optic nerve head (ONH).

Vessel	Time (seconds)^*^		
	POAG	Normal subjects	*P*-value^†^
Cilioretinal artery^‡^	−0.85 ± 0.54	−0.59 ± 0.77	0.617
Central retinal artery	−0.72 ± 0.98	−0.92 ± 0.93	0.555
Parapapillary choroidal arteriole	−0.61 ± 0.69	−0.94 ± 0.85	0.235
Microvessels crossing from the choroid to the ONH	0.00 ± 0.00	0.00 ± 0.00	—
Peak intrapapillary filling	10.46 ± 2.85	9.83 ± 3.43	0.687

Data are mean ± SD values.

^*^Time difference between the initial appearance of ICG dye in each vessel and within the ONH. Negative value indicates that dye appeared prior to the appearance in the ONH.

^†^Calculated using the Mann-Whitney U test.

^‡^Cilioretinal artery was found in five eyes (15.2%) in POAG and four eyes (40.0%) in normal subjects.

### Regional absence of microvessels between the parapapillary choroid and the ONH

**Figure 6:**
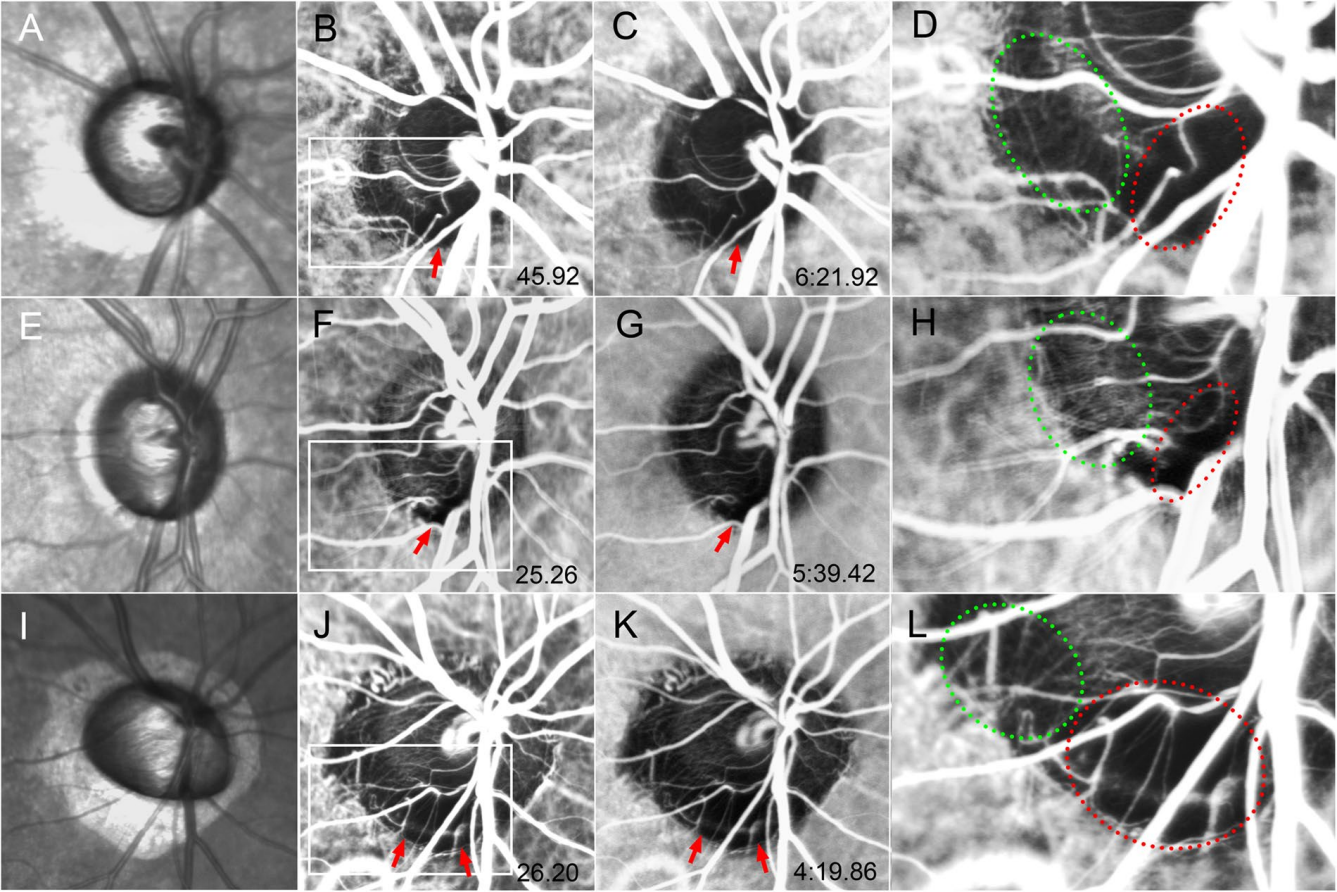
Serial ICGA images of three eyes demonstrating perfusion defects throughout the entire ICGA session. (**A**,**E**,**I**) Infrared fundus images. (**B**,**F**,**J**) ICGA images obtained at the time of peak ONH filing. Perfusion defects are observed (arrows). (**C**,**G**,**K**) Late-phase ICGA images of optic disc filling. Although the image contrast is decreased, perfusion defects are still evident (arrows). (**D**,**H**,**L**) Magnified views of the optic disc marked with the box at the peak phase shown in. (**B**,**F**,**J**) Microvessels supplying the ONH are clearly seen in the area of ONH filling (green dotted area), but they are absent in the area of the filling defect (red dotted area).

Microvessels crossing over the optic disc margin were not visualized regionally throughout the entire ICGA session in 15 POAG eyes (45.5%). Such finding was not observed in any of the healthy eyes. In those regions the corresponding ONH sector was not perfused until the end phase of ICGA. Sectoral nonperfusion was also observed in the corresponding parapapillary area (Fig. 6, Supplemental video 2).

## Discussion

The temporal sequence of first dye appearance in the retinal, choroidal and ciliary artery was diverse among subjects. The CRA, SPCA and cilioretinal arteries are all branches of ophthalmic artery. The interindividual variation of the first dye appearance in those branch vessels reflects variation in the length of the path of blood vessels and in the velocity of the blood flow in those vessels.

An important finding of this study is that the parapapillary choroidal arteriole was universally filled before the adjacent ONH filling. The parapapillary choroid and the prelaminar tissue share the common source of perfusion (i.e. SPCA). If the parapapillary choroid and the prelaminar tissue are supplied by separate branches of SPCA, the time sequence of perfusion between the former and latter may be varying among individuals as is seen in other branches of ophthalmic artery in the present study. However, the dye appeared in the ONH in a sectoral fashion only after the substantial filling of the adjacent choroid in all eyes. These data suggest that the prelaminar tissue is likely to be distal to the parapapillary choroid in perfusion. If not, the branches supplying the parapapillary choroid and the prelaminar tissue should have a comparable length and similar velocity along their paths from their common origin (i.e., more proximal branch of SPCA) in all cases, which is not likely.

The sectoral ONH perfusion was initiated by the appearance of microvessels crossing over the optic disc margin. Moreover, when these vessels were not visible at any time point in the ICGA session, the corresponding sector of the ONH was also not perfused. These finding suggests that these microvessels are directly responsible for the ONH perfusion. The origin of these microvessels is of particular interest. It is obvious that they are not the branches of retinal vessels since their appearance was not related with the dye appearance in the CRA. Then, they can be either choroidal or scleral vessels. It is known that vessels below the choroid are not easily visible on ICGA^20,21^. Scleral vessels such as Zinn Haller circle were reported to be only visible in ICGA in the area of parapapillary atrophy^22^. If the microvessels crossing over the optic disc margin were underneath the choroidal layer, they should be observed more easily through the window of choroidal perfusion defect. This was not the case: the microvessels were not visible in any case of choroidal perfusion defect. Therefore, it may be reasonably proposed that they are in the choroidal layer and dropped out in eyes with choroidal perfusion defect (i.e., MvD). Taken together, our data suggest that ONH perfusion, which occurs in a sectoral fashion, is largely dependent on the centripetal flow from the parapapillary choroid.

Cioffi *et al*. demonstrated small vessels between the parapapillary choroid and ONH using corrosion casting^23^. We consider that the microvessels crossing over the optic disc margin are the correspondent of the small vessels from the parapillary choroid and ONH identified by corrosion casting. Although Cioffi *et al*. asserted that the contribution of these centripetal small vessels to the prelaminar region is minor compared to the direct branches from the SPCA^23^, our data suggest that those microvessels may give a critical contribution to prelaminar tissue perfusion. This notion is consistent with other studies which demonstrated the dependency of ONH perfusion to the adjacent parapapillary choroid in experimental occlusion model of posterior ciliary artery^24^ or in eyes with nonarteritic anterior ischemic optic neuropathy^25,26^.

This study has a limitation. The sample size was small. A larger study is needed to investigate whether there is an interindividual variation in the dependency of ONH blood supply to parapapillary choroid. However, universal dependency of sectoral ONH perfusion on the dye appearance in the adjacent parapapillary choroid and microvessels crossing over the disc margin from the parapapillary choroid suggests that this is likely the predominant variation if variations exist.

In conclusion, we have demonstrated that the perfusion of the ONH sector was dependent on the dye appearance in the microvessels crossing over the optic disc margin from the parapapillary choroid to the ONH. The findings suggest that the centripetal flow from the parapapillary choroid is an important source of anterior ONH perfusion.

## Methods

### Participants

This prospective study enrolled POAG patients who visited Seoul National University Bundang Hospital between November 2014 and May 2015. Healthy control subjects were recruited by advertisement and also among patients who visited the clinic due to nonglaucomatous problem (e.g., incipient cataract, dry eye). Written informed consent to participate was obtained from all subjects. This study was approved by the Seoul National University Bundang Hospital Institutional Review Board and followed the tenets of the Declaration of Helsinki.

All participants underwent comprehensive ophthalmic examinations that included best-corrected visual acuity (BCVA), Goldmann applanation tonometry, refraction tests, slit-lamp biomicroscopy, gonioscopy, dilated stereoscopic examination of the optic disc, disc photography (EOS D60 digital camera, Canon, Utsunomiyashi, Tochigiken, Japan), measurements of the central corneal thickness (Orbscan II, Bausch & Lomb Surgical, Rochester, NY, USA) and axial length (IOL Master version 5, Carl Zeiss Meditec, Dublin, CA, USA), spectral-domain OCT (Spectralis, Heidelberg Engineering, Heidelberg, Germany), and standard automated perimetry (Humphrey Field Analyzer II 750 and 24–2 Swedish interactive threshold algorithm, Carl Zeiss Meditec).

POAG was defined as the presence of an open iridocorneal angle, glaucomatous optic neuropathy such as notching, rim thinning, and RNFL defect, and corresponding defects in the visual field. A glaucomatous visual field defect was defined as (1) outside the normal limits on the glaucoma hemifield test; (2) three abnormal points, with a probability of being normal of *P* < 0.05, and one abnormal point with a probability of being normal of *P* < 0.01 by pattern deviation; or (3) a pattern standard deviation of <5% confirmed on two consecutive reliable tests (fixation loss rate of ≤20%, and false-positive and false-negative error rates of ≤25%). The normal controls had an IOP of ≤21 mmHg, no history of increased IOP, a normal appearing optic disc, and a normal visual field.

To be included, eyes had to have a BCVA of 20/40 or better, a spherical equivalent range from −8.0 diopters to o 3.0 diopters, cylinder correction within ±3.0 diopters, and no history of intraocular or corneal refractive surgery. The exclusion criteria were combined retinal or neurologic diseases that could affect visual function, unreliable visual field tests (a fixation loss rate of >20%, or false-positive or false-negative error rates of >25%). When both eyes were eligible, one eye was randomly chosen for ICGA.

### Indocyanine green angiography

The parapapillary and intrapapillary choroidal circulation was examined using ICGA^27,28^. Unlike fluorescein, ICG does not leak from the choriocapillaris due to its binding affinity for plasma proteins^29,30^, allowing better evaluation of the fine microvasculature. ICGA was performed using a scanning laser ophthalmoscope (Heidelberg Retinal Angiograph-2, Heidelberg Engineering) equipped with eye-tracker function. A 20° image was obtained around the optic disc. Images were obtained at the fastest possible rate (approximately every 1 to 2 seconds) from the time when the dye first appeared until the peak angiographic phase, and then every 5 to 10 seconds thereafter until 2 minutes after ICG injection. After 2 minutes, 6 to 8 more images were obtained approximately every 30 seconds^11^. The pattern of ONH perfusion was investigated by examining the serial ICGA images. In addition, the microvessels directly responsible for ONH perfusion were sought.

### Temporal relationship between the filling of the optic nerve head and other vascular structures

The times when the dye first appeared within the ONH, CRA, parapapillary larger choroidal arterioles, and cilioretinal artery (when present) were recorded by two masked observers (KML and JMK) who were blinded to the participants’ clinical information. These data were used to determine the temporal relationship between the ONH filling and the filling of other vascular structures. The time of the peak ONH filling was also recorded.

### Data analysis

The interobserver agreement of the measurement of filling time in each vascular structure was assessed by calculating ICC. Mann-Whitney U test and chi-square test were used to compare the group differences as applicable. Statistical tests were performed with commercially available software (Stata version 14.0; StataCorp, College Station, TX). Except where otherwise indicated, the data are presented as mean ± standard deviation.

## Supplementary information

Supplementary information file

Supplemental video 1. Full series of ICGA images of a healthy subject with intact ONH perfusion.

Supplemental video 2. Full series of ICGA images of a glaucoma patient with impaired ONHperfusion.

## Data Availability

Data supporting the findings of the current study are available from the corresponding author on reasonable request.
