# Supplementary material for: Anterior Optic Nerve Head Perfusion is Dependent on Adjacent Parapapillary Choroidal perfusion
Source: Sci Rep. 2019 Jul 29;9:10999. doi: 10.1038/s41598-019-47534-5 (PMC6662773; doi:10.1038/s41598-019-47534-5)
Supplement: Supplementary file 1 — Supplementary information file [file 41598_2019_47534_MOESM1_ESM.docx]

**Anterior Optic Nerve Head Perfusion is Dependent on Adjacent Parapapillary Choroidal perfusion**

Kyoung Min Lee, M.D.^1,2*^, Joon Mo Kim, M.D.^3*^, Eun Ji Lee, M.D.^1,4^, Tae-Woo Kim, M.D.^1,4^

^1^Department of Ophthalmology, Seoul National University College of Medicine, Seoul, Korea

^2^Department of Ophthalmology, Seoul National University Boramae Medical Center, Seoul, Korea

^3^Department of Ophthalmology, Kangbuk Samsung Hospital, Sungkyunkwan University School of Medicine, Seoul, Korea

^4^Department of Ophthalmology, Seoul National University Bundang Hospital, Seongnam, Korea

*Correspondence to:*

Tae-Woo Kim, MD

Department of Ophthalmology, Seoul National University Bundang Hospital,

Seoul National University College of Medicine

82, Gumi-ro, 173 Beon-gil, Bundang-gu,

Seongnam, Gyeonggi-do 463-707, Korea

Tel: 82-31-787-7374, Fax: 82-31-787-4057

Email: twkim7@snu.ac.kr

^*^Kyoung Min Lee and Joon Mo Kim equally contributed to the work and therefore should be considered as equivalent authors.

**Supplemental video 1.** Full series of ICGA images of a healthy subject with intact ONH perfusion.

**Supplemental video 2.** Full series of ICGA images of a glaucoma patient with impaired ONH perfusion.
